# Cerebrovascular Disease and Late-Life Depression: A Scoping Review

**DOI:** 10.7759/cureus.77594

**Published:** 2025-01-17

**Authors:** Harris A Kalim, Tahreem Hussain, Maria Colon, Geilynn Fonseca, Atira Shenoy, Harvey N Mayrovitz

**Affiliations:** 1 Medical School, Nova Southeastern University Dr. Kiran C. Patel College of Osteopathic Medicine, Davie, USA; 2 Medical Education, Nova Southeastern University Dr. Kiran C. Patel College of Allopathic Medicine, Davie, USA

**Keywords:** cerebrovascular disease, cognitive impairment, late-life depression, microvascular depression, vascular markers, white matter lesions

## Abstract

Cerebral small-vessel disease (CSVD) is an umbrella term encompassing chronic, progressive conditions that affect the brain’s vasculature. Diverse pathological and neurological factors lead to various clinical and neuroimaging patterns in elderly patients. While depression in the elderly is not uncommon, the connection between CSVD and late-life depression (LLD) remains unclear. CSVD is significant because it is closely linked to chronic hypertension, contributing to microvascular damage and impaired cerebral perfusion. Our objective was to synthesize evidence, evaluate relevant literature to synthesize, and present information relating to the underlying pathophysiology and factors linking CSVD to depression in older adults. Three databases were searched, EMBASE, Ovid MEDLINE, and Web of Science, with the articles selected for inclusion needing to be peer-reviewed, written in English, and published between 1998 and 2022 and have a primary focus on people aged 50 and above who had depression and had a documented history of CSVD. Twenty papers met these criteria and were analyzed, including using statistical correlation. Of the 20 studies, 15 reported a statistically significant correlation between CVSD and LLD, whereas five of the studies found no significant correlation. In the 15 studies that reported a significant relationship between CSVD and LLD, there were a total of 15,158 participants, or an average of approximately 1,011 participants per study. The five studies that did not find a correlation included 2,222 participants, averaging about 444 participants per study. Thus, this review's overall findings are consistent with a significant relationship between CSVD and LLD. White matter hyperintensities (WMHs), one of the findings of CSVD, were found to be a common finding in patients with CSVD and LLD. Increased WMH volume led to an increase in depressive symptoms. However, some studies highlight counterpoints, emphasizing the complexity of the relationship and the influence of non-vascular factors such as neuroinflammation, neurodegeneration, and systemic comorbidities. These findings underscore the importance of early detection of CSVD and interdisciplinary approaches to mitigate the burden of depression and cognitive decline in aging populations. Future research should focus on advanced neuroimaging, genetic profiling, and longitudinal studies to unravel the multifaceted mechanisms linking CSVD and LLD and improve clinical outcomes.

## Introduction and background

Cerebral small-vessel disease (CSVD) refers to a group of chronic and progressive intracranial vascular conditions resulting from diverse pathological and neurological factors [1]. This umbrella term represents a syndrome characterized by various clinical expressions and neuroimaging patterns stemming from structural alterations, such as white matter hyperintensity (WMH) or lacunar infarcts, in the vasculature and brain tissue [1]. Vascular depression (VD) refers to the link between cerebrovascular disease and an increased risk of depressive syndrome in older adults. VD is characterized by persistent executive dysfunction and resistance to antidepressants, whereas late-life depression (LLD) is defined as depression occurring in adults over the age of 60 with no prior episodes of depression before this age [2]. It has been reported that depression in the elderly is not an uncommon occurrence, with an estimated prevalence of 13.3% [3]. However, the precise role and impact of CSVD in depression among the elderly have not been fully understood. Thus, there exists a gap in our understanding of the underlying pathophysiology behind the connection between CSVD and LLD.

Diagnosis

CSVD is diagnosed using magnetic resonance imaging (MRI) biomarkers, including lacunar infarcts, cerebral microbleeds, WMHs, and cerebral atrophy [1]. WMHs are the most common imaging biomarker indicative of CSVD, as white matter is an area of the brain responsible for transmitting information to other areas. Therefore, any abnormality in this tissue can result in communication errors among brain regions [4]. WMH is usually found as an incidental finding in asymptomatic cases. The participants in the studies included in this scoping review were screened for these markers of CSVD. Studies utilized T2-weighted and fluid-attenuated inversion recovery (FLAIR) MRI to characterize WMHs, with some additionally using diffusion tensor MRI (DT-MRI) and magnetization transfer MRI (MT-MRI) to distinguish WMHs from other lesions [1]. Other studies employed CT angiography and CT perfusion to identify depression-related decreases in cerebral blood flow (CBF) and microcirculatory changes, such as lacunar infarcts and cerebral microbleeds [5]. These diagnostic measures allow for a thorough brain anatomical distribution and functioning analysis. Hemodynamic changes that lead to decreases in CBF have been considered to play a role in CSVD, as cerebral microbleeds cause vascular damage to areas of the brain, controlling affect and cognition, such as in the prefrontal cortex [6]. Brain atrophy, particularly in regions affected by small-vessel disease, may further impair cognitive and emotional regulation due to ischemic processes. Additionally, enlarged Virchow-Robin spaces (VRSs) are considered markers of small-vessel disease and suggest microvascular brain damage, particularly in the basal ganglia. They serve as clinically significant indicators of cerebral microangiopathy and aid in differentiating subtypes of CSVD, such as those associated with LLD [7]. Decreased CBF, increased WMH volume, and cortical and central atrophy contribute to a wide range of functional brain deficiencies, exacerbating depressive symptoms in patients [6,8].

Vascular connections

Vascular markers, alongside MRI markers, can indicate the presence of CSVD. Vascular cell adhesion molecule-1 (VCAM-1), an endothelial serum biomarker using enzyme-linked immunosorbent assay (ELISA), is indicative of vascular inflammation and can be linked to endothelial dysfunction, serving as a link between CSVD and its causative effects rather than a diagnostic tool [9]. Vascular impairments, including reduced blood vessel response and increased carotid intima-media thickness (CIMT) caused by atherosclerosis, can be used as markers for small-vessel disease in the brain [7]. In addition to direct vascular markers, specific peripheral markers can reflect broader systemic changes associated with CSVD. Peripherally, increased arterial stiffness in the carotid-femoral arterial segment may play a role. One measure used to assess this is the carotid-femoral pulse wave velocity (CFPWV). A higher pulse wave velocity means the blood pressure pulse wave moves more quickly through the arteries, indicating the arteries are stiff. When arteries are stiff, they lose some of their ability to absorb the pressure from blood pumped by the heart, which increases the strain on smaller blood vessels, such as those in the brain. This added strain can lead to microvascular damage and affect brain function [10]. Finally, CSVD is often considered a diagnosis of exclusion. Using medical and neuropsychiatric examinations, several studies ruled out brain injury, organic brain disease, and lifetime substance use as causes of disruption to cerebral hemodynamic and endothelial dysfunction [8,11-13].

Study goal

This review aims to bridge the knowledge gap by creating the first comprehensive conceptual map by synthesizing imaging, vascular, and clinical data of the currently predicted pathological causes of depression in elderly individuals with a history of CSVD, with a focus on the potential role of CSVD and its impacts on brain function. CSVD and LLD will be broken down into subsections including general aspects and specific correlations between the two that allow us to bridge and organize the ideas in various papers. WMHs, indicative of cerebrovascular disease, are found in up to 80% of healthy 60-year-olds and 95% of people aged 90 and older, highlighting the prevalence and increasing risks with age [4]. Given the rising rates of depression and cerebrovascular disease globally, particularly among elderly populations, there is a pressing need for a clearer understanding of the role that small-vessel damage in the brain may play in triggering or exacerbating depressive symptoms, which has been underrepresented in current research [14]. LLD is of great interest because of its complex basis and its outcome in the depressed elderly, increasing the risk of cognitive impairment and poor quality of life [15].

## Review

Methods 

Search Strategy 

A base search strategy was constructed by an analysis of key terms from relevant articles in EMBASE, Ovid MEDLINE, and Web of Science. All searches were conducted in September 2023, and the base search was utilized for each database. To be included in the scoping review, articles need to be peer-reviewed, written in English, and published between 1998 and 2022 and have a primary focus on people who had depression, were at least 50 years old, and had a history of CSVD. Excluded articles were other scoping or systemic reviews, dissertations, books, recommendations, opinions, policies, or guidelines. Articles with a primary focus on neuropsychiatric manifestations in patients with CSVD were also excluded.

Identification and Selection of Studies 

**Figure 1:**
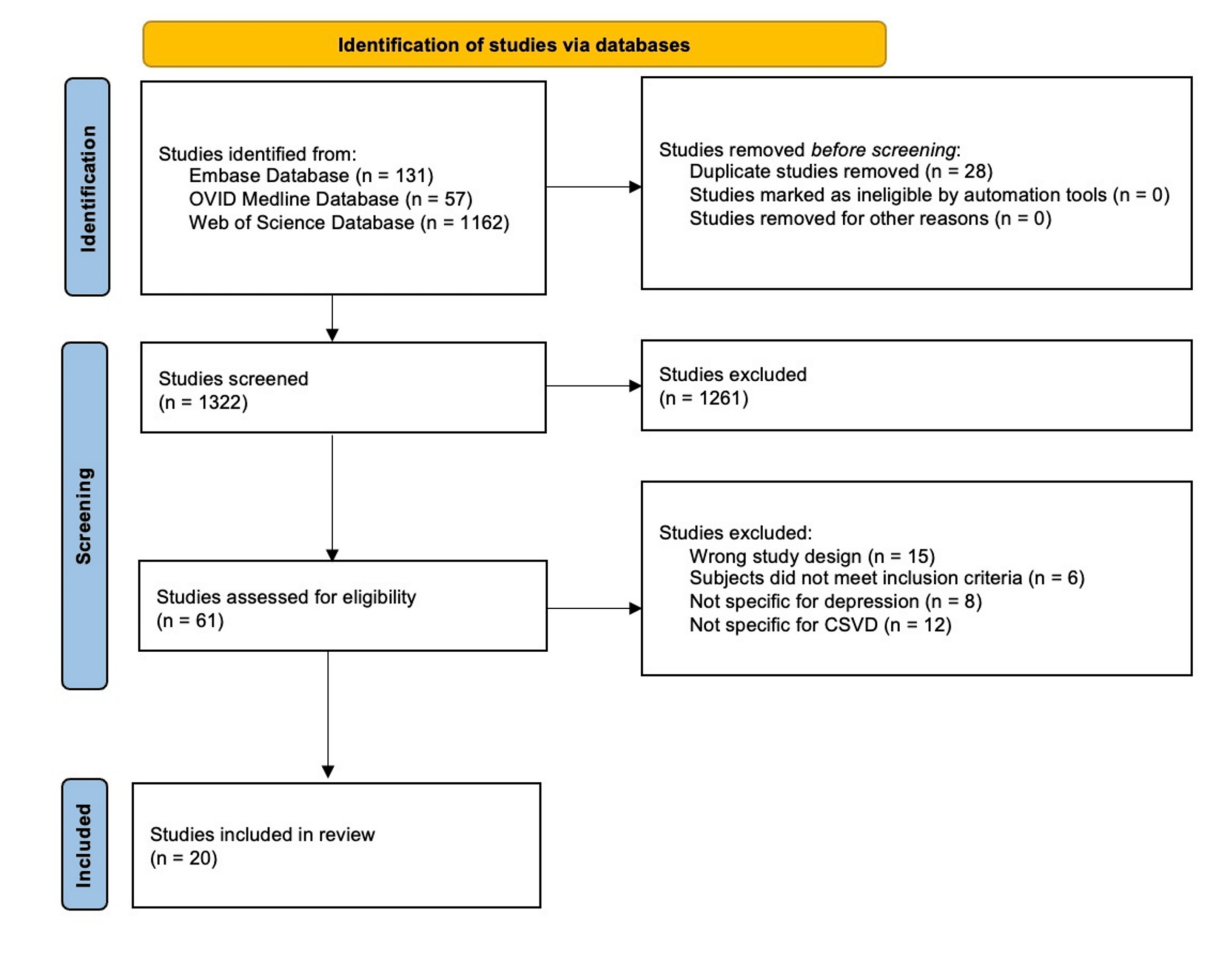
Search approach. Diagram showing the Preferred Reporting Items for Systemic Reviews and Meta-Analyses (PRISMA) flowchart

The text words contained in the titles and abstracts of relevant articles and the index terms used to describe the articles were used to develop a complete search strategy for three databases (EMBASE, Ovid MEDLINE, and Web of Science). The following defined search terms were used: (“cerebrovascular small vessel disease” OR “microvascular dysfunction”/exp OR “microvascular dysfunction”) AND (“vascular depression”/exp OR “vascular depression” OR “depression” OR “cognitive impairment”/exp OR “cognitive impairment” OR “white matter lesions”) AND (“older adults”/exp OR “older adults” OR “elderly”/exp OR “elderly” OR “geriatric”/exp OR “geriatric” OR “aged”/exp OR “aged”). The search strategy, including all identified keywords and index terms, was utilized for each included database, as shown in Figure 1.

From a search of three databases, 1,350 published articles were identified, as outlined in Figure 1. After removing duplicates, 1,322 titles and abstracts were screened by two reviewers, and a third reviewed any conflicts between the first two reviewers through round table discussions regarding differing views. After the first screening, 1,261 articles were excluded, and all authors assessed the 61 articles according to the inclusion and exclusion criteria. Based on the criteria, 41 articles were excluded, and thus, 20 articles were included in the final review. Of the 20 articles, six were case-control studies, 11 were cohort studies, one was a retrospective analysis, and two were cross-sectional studies. Emphasizing the significance of article quality and validity within the article selection process is essential. The screening process of the 20 included studies involved the utilization of a critical evaluation tool, specifically Joanna Briggs Institute Critical Appraisal Tools, to assess for risk of bias. Consequently, all 20 articles were retained for the final scoping review. This enabled a detailed discussion on the relevance and quality of each article within the research context.

Rayyan (Rayyan Systems Inc., Cambridge, MA, US) and Excel (Microsoft Corp., Redmond, WA, US) software programs were used to manage data for this review, encompassing participant details, study context and concept, methodologies, and key findings relevant to the review [16]. The Preferred Reporting Items for Systemic Reviews and Meta-Analyses (PRISMA) diagram template was obtained from the Equator Network’s PRISMA Flow Diagram website. Data compiled in Rayyan and Excel was then entered into the PRISMA diagram, which was finalized based on the established inclusion and exclusion criteria. The studies were organized by factors such as aim, sample size, study type, and principal conclusions, as presented in Table 1.

**Table 1:** Summary table of the included articles WMH: white matter hyperintensities; MRI: magnetic resonance imaging; DWMH: deep white matter hyperintensities; LOD: late-onset depression; VCAM-1: vascular cell adhesion molecule-1; MCA: middle cerebral artery; MD: mean diffusivity; CI: cognitive impairment

Reference	Country	Study aim	Sample size	Study design	Findings
Ali et al., 2023 [4]	Germany	To examine the relationship between specific cerebrovascular risk factors and the extent of WMH and how this can influence the occurrence of recurrent vascular events, CI, and depression in patients who have experienced a first ischemic stroke	431	Retrospective analysis	There is no distinct correlation observed between the specific risk factors and the extent of WMH due to various limitations such as small sample size and limited data on prestroke medication use and previous cognitive impairment
Wang et al., 2020 [5]	China	To investigate the relationship of LOD with macrovascular and microvascular changes in the brain by using a multi-imaging method, including computed tomography angiography, CT perfusion, and MRI	116	Case-control study	A decreased global or regional perfusion state, moderate-to-severe stenosis of MCAs, and increased WMH loads could be used as imaging biomarkers to suggest diffuse or localized cerebral macrovascular and microvascular pathology in LOD
Puglisi et al., 2018 [6]	Italy	To investigate transcranial Doppler ultrasound parameters with cognitive function and the intensity of subcortical ischemic vascular disease	76	Cross-sectional study	In patients with late-life depression, hemodynamic impairment may play a role in the development of cognitive decline
Paranthaman et al., 2010 [7]	United Kingdom	To determine the connection among endothelial function, arterial stiffness, and atherosclerosis in vascular beds in elderly patients with depressive disorder	46 (25 patients + 21 controls)	Case-control study	In older adults, depression is linked to reduced endothelial function and increased atherosclerosis
Jokinen et al., 2009 [8]	Finland	To characterize the clinical and cognitive features of MRI-defined subcortical ischemic vascular disease in a diverse group of functionally independent older adults with white matter lesions	613 (89 patients + 524 reference patients)	Longitudinal cohort study	Subcortical ischemic vascular disease was correlated to certain clinical and functional attributes
Tchalla et al., 2015 [9]	United States of America	To investigate whether increased levels of VCAM-1 is a marker of depressive symptoms in relation to cerebral vascular disease	680	Prospective cohort study	Increased VCAM-1 and its relationship to symptoms of depression may be due to endothelial dysfunction from cerebral microvascular damage
van Sloten et al., 2016 [10]	Netherlands, United States of America	To evaluate if arterial stiffness is correlated with symptoms of depression and if cerebral small-vessel disease is also a contributing factor	2,058	Cross-sectional cohort study	Greater arterial stiffness is correlated with an increase in depressive symptoms; this association is partially explained by white matter hyperintensity volume and subcortical infarcts
Dalby et al., 2021 [11]	Denmark	To investigate capillary function in individuals with late-onset major depressive disorder	44 (22 patients + 22 controls)	Case-control study	Alterations in perfusion within the deep nuclei and cerebellum indicate unusually low and high levels of activity in patients with major depressive disorder
Lyness et al., 1998 [12]	United States of America	To assess and compare the impact of systemic cerebrovascular risk factors in individuals with major depression	194 (130 patients + 64 controls)	Case-control study	Did not provide evidence to support the idea that a linear model of small-vessel disease is applicable to most older patients with major depression due to cerebrovascular risk factors not having a significant association with depression variables and the following limitations: small sample size and young age of patients
Lee and Kim, 2020 [13]	South Korea	To explore the connections between white matter hyperintensities, depressive symptoms, and cognitive function and to investigate how depressive symptoms mediate the relationship between WMH and cognitive impairment	1,158	Cross-sectional study	The model that most accurately represented the relationships among the variables showed that DWMH influenced cognitive function both directly and indirectly via depressive symptoms
Özel et al., 2023 [14]	Sweden, Netherlands, Singapore, Turkey	To assess the relationship between neuroimaging markers and symptoms of depression	4,943	Population-based cohort	Symptoms of depression were correlated with neuroimaging markers of white matter microstructural damage such as lacunar infarcts, WMH, brain tissue segmentation, and MD. In particular, MD association with depressive symptoms was statistically significant
Direk et al., 2016 [26]	Netherlands	To investigate the relationship among white matter lesions, lacunar infarcts, cerebral microbleeds, and the spectrum of depression	3,799	Prospective cohort study	White matter lesions and lacunar infarcts can be non-specific vascular abnormalities observed in cases of depression
Lyness et al., 1999 [28]	United States of America	To investigate whether cerebrovascular risk factors are correlated with a depression diagnosis and symptoms	303	Case-control study	The data did not endorse that a small-vessel brain disease model of depression can apply to older adults due to the limited statistical power of the study
Wei et al., 2018 [19]	China, United States of America	To assess cerebral blood flow changes throughout antidepressant treatment and the impact of these changes on depressive symptoms	46	Longitudinal cohort study	Regional cerebral blood flow increases were correlated with a decrease in symptoms of depression
Greenstein et al., 2010 [20]	United Kingdom	To investigate the structure and function of small subcutaneous arteries in individuals with late-life depression	31 (16 patients + 15 controls)	Case-control study	Depressed patients with cerebral microvascular damage exhibit abnormalities in the structure and function of small subcutaneous arteries, even when their cardiovascular profiles are similar
Sibolt et al., 2013 [21]	Finland	To determine whether patients with poststroke depression or depression-executive dysfunction syndrome exhibit higher rates of stroke recurrence	223	Cohort study	Depression and depression-executive dysfunction syndrome are linked to a shorter time to recurrence of ischemic stroke and a faster rate of stroke recurrence
Lamar et al., 2010 [25]	United Kingdom	To determine whether white matter damage in euthymic older adults is associated with experiencing depressive symptoms	79	Population-based cohort	A link has been identified between white matter damage and the presence of depressive symptoms in euthymic older adults
Tully et al., 2017 [27]	France, Australia, United States of America	To determine WMH volume regarding specific depressive symptoms over 10 years	1,440	Prospective longitudinal cohort study	Regional WMH volumes and certain depressive symptoms have clinical and prognostic relevance to help discern between persons at risk for depression and dementia
Mewton et al., 2019 [29]	Australia, United Kingdom	To determine the association among depression symptoms, cognition, and cerebrovascular disease	462	Prospective cohort study	Determined that depressive symptoms forecast cognitive decline independently of vascular disease
Grool et al., 2013 [30]	Netherlands, Sweden	To determine the impact lacunar infarcts and white matter lesions have on the intensity and course of depressive symptoms	650	Cross-sectional cohort	Lacunar infarcts were not correlated with the intensity or the course of depressive symptoms

General aspects of LLD associated with CSVD 

Through diagnostic imaging, some literature indicates a strong understanding that ischemic changes caused by CSVD were evident in patients who developed LLD. The literature suggests that the chronic and progressive manifestations of CSVD cause ischemic changes in the brain, which leads to white matter lesions (WMLs) and, ultimately, depression and cognitive impairment. It has been hypothesized that there could be genetic mutations linked to the predisposition of some individuals developing CSVD, which will lead to LLD. Other studies focused on factors aside from WMLs, including environmental and genetic risk factors that may play a role in both CSVD and depression.

Correlations between LLD and CSVD 

Neuroimaging and Biological Markers 

When assessing the correlation between LLD in persons with CSVD, 15 studies reported a positive association based on varying neuroimaging and biomarkers. Interest has been expressed in genetic polymorphisms that may increase the risk of developing hyperintensities, including those related to blood pressure or homocysteine metabolism. One such polymorphism, the brain-derived neurotrophic factor (BDNF) Val66Met allele, was found to be associated with hyperintense lesions in older individuals [17]. The same allele was more commonly seen in depressed individuals as well. The BDNF Val66Met polymorphism is associated with altered intracellular processing and secretion of BDNF, potentially impairing neuroprotection. In the context of cerebrovascular disease and LLD, this polymorphism, particularly the Met66 allele, is linked to greater WMH volumes, often indicative of ischemic damage. BDNF has been shown to protect neurons against ischemic injury, and the Met66 allele may reduce this protective effect, exacerbating brain damage. Furthermore, the Met66 allele is more prevalent in individuals with LLD, possibly due to its influence on neurogenesis and structural changes in brain regions critical for mood regulation, such as the hippocampus and prefrontal cortex.

A study of 680 subjects aged 72-83 found an association between depressive symptoms and elevated serum levels of the biomarker VCAM-1 [9]. The study used the Revised Center for Epidemiologic Studies Depression Scale (CESD-R) to determine whether participants exhibited depressive symptoms. In the group with depressive symptoms, the mean VCAM-1 serum concentration was 1,239 ng/mL as opposed to 1,177 ng/mL in the group without depressive symptoms (p = 0.04). These subjects also had statistically significant greater incidences of vascular conditions, including hypertension (HTN), coronary artery disease, and hyperlipidemia, although a diagnosis of CSVD was not explicitly specified. VCAM-1 is an endothelial protein that can help direct white blood cells to injury sites. Thus, elevated levels of VCAM-1 in patients with CSVD are to be expected. Due to mood impairment from damage to the brain’s frontal-subcortical circuits, it was hypothesized that VCAM-1 can be a crucial marker for LLD secondary to CSVD [9].

A study of 4,943 participants used neuroimaging markers such as total brain volume, gray matter volume, white matter volume, WMH volume, cortical infarcts, lacunar infarcts, microbleeds, fractional anisotropy, and mean diffusivity. These markers were used to determine that smaller total brain volume, including decreased white matter volume, was correlated with the LLD hypothesis [14]. The study’s patients had a broad age range (64.6 ± 11 years) and included both sexes, different education levels, and comorbidities including diabetes, HTN, and heart diseases, as well as smoking status and alcohol consumption. Furthermore, a case-control study of 116 subjects hypothesized that WMH, a low perfusion state, or stenosis of the middle cerebral artery could be used as neuroimaging markers to indicate LLD [5]. The results of this study showed that the depressed group, ranging in age from 72.5 ± 9.4 years, was more prone to intracranial stenosis than the control group, ranging in age from 69.1 ± 9.1 years (p < 0.05).

Cerebral Vascular Aspects

Cerebrovascular risk factors and aspects of vascular function have been compared to the incidence of depressive symptoms in elderly patients. The existence of numerous cerebral microbleeds, especially those in multiple brain lobes, is linked to an increased overall burden of neuropsychiatric issues, notably depression and disinhibition [1]. This study found that when small blood vessels lose their capacity to regulate blood flow efficiently, it can lead to chronically reduced CBF leading to the formation of lacunar infarctions from arterial blockage-induced severe ischemia [1]. The accumulation of amyloid due to angiopathy has also been seen in patients with depression in studies looking at intracerebral hemorrhaged survivors [18].

In one study, 76 depressed patients, ages 72.5 ± 5.3 years, had comorbidities such as HTN, diabetes, and dyslipidemia, which impacted blood flow in the brain and eventually caused vascular damage, inducing the incidence of depressive symptoms [6]. The study aimed to investigate cerebral hemodynamics in VD, focusing on the relationship between transcranial Doppler (TCD) parameters, WMLs, and cognitive and depressive symptoms in elderly patients. Depressive symptoms were measured using the Hamilton Depression Rating Scale, and cognitive function was evaluated via the Stroop Color-Word Test. Key findings revealed that increased pulsatility index and reduced mean blood flow velocity were associated with severe WMLs, depressive symptoms, and executive dysfunction. The study highlights cerebral hemodynamic dysfunction as a marker for VD.

Another study of 44 subjects, ages 57.4 ± 4.6 years, had 22 subjects with LLD who showed depressive symptoms after the age of 50, focused on the role of capillary dysfunction in LLD and its effects on CBF, cerebral blood volume (CBV), oxygen use, and capillary transit-time heterogeneity (CTH) [11]. This study looked at how capillary dysfunction affects brain blood flow and oxygen use in people with LLD by measuring factors such as CTH, which is the time it takes blood to flow through capillaries in a specific area of the brain, using advanced MRI. They found impaired blood flow regulation in key brain regions linked to mood and cognition, reduced oxygen use in some subcortical areas, and increased activity in the brainstem and cerebellum. They also found evidence suggesting that reduced capillary density and perfusion in the left orbitofrontal cortex (OFC) and frontal cortex may be associated with increased frequency or severity of depressive episodes emphasizing the importance of understanding small blood vessel health in treating depression and preventing brain degeneration [11]. A longitudinal study of 46 individuals with LLD explored changes in CBF during antidepressant treatment using arterial spin labeling MRI. It found that increased regional CBF, particularly in areas like the cingulate cortex, correlated with reductions in depressive symptoms over 12 weeks. This supports the VD hypothesis by validating that impaired blood flow correlates with depressive symptoms in patients [19]. However, these small sample sizes limit the generalizability of findings by increasing the risk of selection bias and random error, making it difficult to extrapolate results to broader populations.

Small-artery structures including the effects of increased atherosclerosis, arterial stiffness, and endothelial function (EF) have been found to play a role in vascular dysfunction in LLD patients. In a study of older adults with LLD, 16 depressed patients (aged 71.8 ± 4.0 years) were compared to 15 healthy controls (aged 72.1 ± 5.9 years) to explore how small-artery abnormalities contribute to cerebrovascular damage and depression [20]. Depressed patients exhibited reduced EF, measured as vasodilation in response to acetylcholine, at 84.0% compared to 96.0% in controls (p < 0.03), despite many being on medications like statins that typically improve vascular health. Structural analysis revealed hypertrophy, or thickening of arterial walls, with depressed patients showing significantly greater wall thickness and cross-sectional area compared to controls (p < 0.04). Furthermore, these patients had more dilated basal ganglia VRSs, a marker of microvascular damage, though WML scores were similar between groups. These findings suggest that vascular dysfunction, characterized by impaired EF, arterial stiffness, and microvascular damage, plays a significant role in LLD, supporting the VD hypothesis and highlighting the potential benefits of vasoprotective therapies. Inflammation and oxidative stress play more pronounced roles in LLD than in early-onset depression, likely due to aging-related vulnerabilities and overlap with cerebrovascular conditions.

Peripheral Vascular Aspects

The relationship between LLD and arterial stiffness was assessed using multiple factors including CFPWV, CIMT, and hormone levels. Carotid-femoral velocity measures arterial stiffness by determining the speed at which blood flows from the carotid to femoral arteries and finding that a higher CFPWV indicates greater arterial stiffness [10]. CFPWV is an important marker of vascular health, and increased CFPWV indicates stiffened arteries, often caused by aging, HTN, or atherosclerosis, and is associated with greater cardiovascular risk and end-organ damage. Arterial stiffness impairs cerebral perfusion and damages small vessels, contributing to WMHs and ischemic changes in the brain that are linked to depression. Depression and arterial stiffness share common risk factors, such as systemic inflammation and HTN. Measurements of arterial stiffness were determined via CFPWV in 2,058 subjects, with an average age of 79, indicating that an increase in arterial stiffness is correlated with more depression symptoms [10].

Another way in which the relationship between LLD and arterial stiffness was assessed included the measurement of CIMT. CIMT allows us to assess plaque building up between the intima and medial layers of the arteries, a sign of the damage caused to arteries by atherosclerosis. Increased CIMT is associated with reduced cerebral perfusion and heightened risk of cerebrovascular disease, which are central to the hypothesis that vascular changes contribute to the onset or exacerbation of depression. These structural alterations in carotid arteries may lead to WMHs and other brain changes linked to depressive symptoms, particularly in older adults. A case-control study of 25 patients, which involved participants aged 60 years and older, with a mean age of 72.4 years, revealed that impaired EF, resistant vessels, and increased atherosclerosis were found to be higher in elderly patients with depressive symptoms [7]. The research assessed these factors via CIMT, dose-response to acetylcholine from gluteal fat biopsies of resistance vessels, and brain MRI scans. Depressed subjects showed significantly reduced maximal vasodilation in response to acetylcholine in small arteries. This indicates impaired EF, a key step in atherosclerosis development. Reduced EF in peripheral vessels suggests systemic vascular dysfunction, which may extend to cerebral vessels. The findings revealed significant vascular dysfunction and structural brain abnormalities in older adults with depression, supporting the "vascular depression" hypothesis and highlighting the potential role of vascular impairment in its development.

The evaluation of stress hormones was also used to assess vascular functioning as stress hormones are associated with dysfunction of the immune defense systems, endothelial damage, increased platelet aggregation, and clotting cascade activation [21]. A study involving 223 poststroke patients demonstrated that depression, particularly depression-executive dysfunction syndrome (DES), is associated with an elevated risk of recurrent ischemic stroke due to increased tonic levels of stress hormones. DES is closely associated with dysfunction in frontostriatal pathways and white matter changes, often seen in vascular-related conditions. DES differs from other depressive subtypes in that DES is characterized by impairments in sequencing, organizing, planning, abstracting, psychomotor function, and a general reduction in interest in activities. DES patients, which tend to be older individuals than non-DES patients, exhibit worse long-term outcomes, including higher recurrence of strokes and greater overall morbidity and mortality. A combination of medications, including statins, antihypertensives, antiplatelet agents, and appropriate antidepressants, could integrate depression management into stroke prevention. Additionally, the supplemental use of antioxidants, either through diet or medication, may be beneficial. The cumulative stroke recurrence risk was higher in depressed patients during a 12-year follow-up, emphasizing the impact of neuropsychiatric symptoms on cerebrovascular health. This underscores the need for integrating depression management into secondary stroke prevention [21].

White Matter Lesions

One prevailing theory posits that WMLs may arise from ischemia. Alterations in blood flow dynamics, specifically impaired CBF autoregulation, could be a central player in this ischemic process [22]. Mild loss of perfusion with age is normal; however, patients who were depressed may have had a lower threshold, which made them more vulnerable to ischemia. Hyperintensities are more severe and of greater volume in older depressed subjects than in comparison groups. This increased hyperintensity severity and volume is associated with poorer antidepressant response and a more chronic course of depression [17]. According to a study, there was a statistically significant positive correlation between WMLs of ischemic natures in depressed patients compared to the control group [23]. The pathophysiology of deep WMHs (DWMHs) in elderly subjects with severe depression was explored further, and ischemic changes were commonly found within the dorsolateral prefrontal cortex (DLPFC) [23]. Study results were consistent with the VD hypothesis, which holds that WMLs are caused by cerebrovascular disease disrupting fiber tracts within frontostriatal circuits [24].

WMLs are typically found in patients with prior ischemic events. In a study with 79 participants, with an average age of 68 years, diffusion tensor imaging and MRI concluded that there was an association between WMLs and depressive symptoms [25]. Furthermore, in 89 elderly patients, subcortical changes were correlated with clinical symptoms of depression [8]. Conversely, another study of 3,799 subjects found that WMLs or infarcts may be non-specific findings seen in depression [26]. WMHs are common in older adults and associated with various conditions, including depressive symptoms, major depressive disorder, anxiety, and dementia. This broad association suggests they are not unique to depression. The relationship between WMH and depressive symptoms is more evident in specific subcortical regions, such as the frontostriatal and limbic areas, which are crucial for mood regulation. WMHs are linked to CSVD resulting from HTN, arteriolosclerosis, inflammation, or amyloid deposition. These conditions are prevalent in aging populations, contributing to the non-specific nature of WMH concerning depression. Cognitive functioning was a variable used to assess the role of WMH. Both DWMH and periventricular WMH as well as their effects on depressive symptoms were investigated, and DWMHs were found to have a direct and indirect effect on depressive symptoms. In contrast, periventricular WMH only affected cognitive functions [13]. Additionally, regional WMH, both deep and periventricular, can help assess which patients would be at risk for depression and dementia, finding that patients with an increased volume of the WMH were found to have increased depression affect or anhedonia [27].

Environmental and Genetic Risk Factors’ Role in CSVD and Depression

Elevated homocysteine levels, resulting from genetic or dietary deficiencies, have been linked to vascular conditions, but their role in depression remains to be fully confirmed and explored. Elevated homocysteine may contribute to endothelial dysfunction, oxidative stress, and inflammation, which are central to CSVD and neurovascular damage. Similarly, changes in cytokines and activation of the hypothalamic-pituitary-adrenal (HPA) axis occur in vascular dysfunction and could potentially contribute to depression, particularly under conditions of elevated stress.

Hypercortisolemia, a hallmark of HPA axis dysregulation, is associated with both depression and vascular injury [4]. Mechanisms such as increased oxidative stress, impaired endothelial nitric oxide production, and heightened inflammatory responses exacerbate vascular injury. For instance, elevated cortisol levels can induce endothelial apoptosis, weaken the blood-brain barrier, and promote WMH. Emerging evidence suggests a complex interplay between inflammatory cytokines and the HPA axis, where chronic inflammation drives cortisol secretion, creating a feedback loop that worsens both CSVD and depressive symptoms.

The linkages between these inflammatory and endocrine systems, however, remain incompletely mapped, limiting the potential for targeted therapeutic approaches. Understanding these mechanisms could lead to interventions such as dietary folate and vitamin B12 supplementation to address elevated homocysteine levels or pharmacological agents that modulate the HPA axis and reduce inflammation. Future research should focus on identifying biomarkers or using advanced imaging techniques to clarify these pathways, ultimately enabling tailored strategies to reduce the burden of CSVD and its associated neuropsychiatric manifestations.

*Inconclusive Findings* 

Four studies concluded that their data did not support the hypothesis that CSVD was correlated to LLD [3,12,28,29]. The lack of robust control groups and small sample sizes limited statistical power to detect meaningful associations between cerebrovascular risk factors and depression. Some of the studies were unable to confirm a clear association between WMH and CSVD due to the small sample size; however, they were able to support the CSVD hypothesis [3]. One of the studies stated that while their findings did show lacunar infarcts being associated with higher depressive scores, they differed from the VD hypothesis in that their findings were not related to major depressive disorders [30]. Heterogeneity in study populations further complicated interpretations. For instance, one study examined participants with symptomatic atherosclerotic disease, and other studies focused on general primary care populations, creating variability in vascular risk profiles that could influence outcomes. Differences in the diagnostic criteria for depression and variability in the measurement of CSVD markers, such as WMH and lacunar infarcts, also introduced inconsistencies across studies. Another study differed in that it found depressive symptoms may predict cognitive decline, but this is independent of vascular disease itself [29].

Discussion 

*Main Findings of the Review* 

This scoping review aimed to evaluate the extent to which the topic of CSVD affecting LLD has been covered. Our search results generated 20 articles with varying opinions on the cause-and-effect relationship. While 15 out of the 20 articles shared the opinion that damage associated with CSVD aids in leading to LLD, five articles did not support the notion. These five articles did not find a strong enough association but did not entirely reject it [8,12,28-30].

Potential Causative Factors for LLD

The review question that we were looking to answer has been explored over the past few decades, and many papers have shared their varying opinions as to what leads to LLD. Among the topics investigated was the occurrence of WMLs resulting from ischemia [11]. Hemodynamic dysfunction factors, such as low perfusion and high vascular resistance, are a potential causative mechanism in which LLD may precipitate. One paper further listed VCAM-1 as a presumed marker of endothelial activation and dysfunction and concluded that VCAM-1 could potentially be used as a biomarker for cerebrovascular causes of depression [9]. This provided an excellent implication for further research. These results emphasized a robust association between vascular disease and depression rather than an association by clinical risk factors alone.

Each paper was unique in what they determined were variables that could cause depression in the elderly. A different method of looking at causative variables for depression in elderly persons included looking at neuroimaging markers. Factors such as smaller total brain volume, smaller white matter volume, and reduced integrity of white matter were consistently related factors for having depressive symptoms in elderly individuals over time [14]. WMHs were mentioned as a key measurement and predictor of VD, with the extent of LLD dependent on the volume of WMH a patient has [27].

Counterpoints 

There were, however, some studies that did not share the same findings. Some outright denied the causative relationship, and others called for more research and evidence, stating that the relationship cannot be concluded at the current time of publication. These studies called for more research on the vascular mechanisms underpinning VD [29]. With a substantial sample size, a study found that neither WMH volume nor a history of stroke/transient ischemic attack (TIA) predicted an increase in depressive symptoms over time, and no statistically significant data was extracted between sample groups [29]. One study's findings suggest that the idea of depression being linked to small-vessel brain disease might not be relevant for most older individuals experiencing depression symptoms [28]. This was due to their studies being cross-sectional, posing a limitation since the theoretical model suggests that cardiovascular risk factors contribute to depression through the gradual progression of small-vessel brain disease over time. Some other studies indicated that the fluctuating nature of these symptoms, rather than their stability, highlights the importance of conducting repeated assessments of depressive symptoms throughout the follow-up period. This perspective suggests that the relationship between LLD and CSVD may not be significant [30].

Implications for Future Research

We can recommend many implications for future research. These include more significant sample sizes, international studies, and suggestions for preventative measures and therapies for CSVD-causing LLD. Some biomarkers, such as VCAM-1, will require longitudinal studies with robust evidence to be used clinically. As neuroimaging and radiological techniques rapidly advance, it will be intriguing to see what the future may hold regarding more detailed images and statistics on variables such as CBF, lesion sizes, and brain regions affected.

There is also a basis to see what other variables can be affecting results in an elderly population prone to multiple morbidities. Many elderly patients diagnosed with LLD will also be taking multiple medications for these morbidities. An example of medications that can alter results include antidepressants, which have been shown to influence capillary function and CBF, whether it be positive or negative [8]. There could be an implication to see the effect of various antidepressants and drugs on the results of VD in the elderly. These complex and varying interactions between morbidities and their treatments add to the complexity of discovering the exact pathophysiology of this topic. For future research, refining diagnostic criteria for depression subtypes, such as VD, could help clarify the relationship between CSVD and depressive symptoms. Longitudinal studies are needed to determine whether CSVD precedes or exacerbates depression and to identify potential mediators, such as inflammation or neurochemical disruptions. Additionally, studies should aim to reduce population heterogeneity by focusing on specific groups, such as older adults with defined vascular profiles, to improve consistency. Alternative hypotheses, including overlapping mechanisms like neurodegeneration and metabolic dysfunction, should also be explored. Clinically, integrating imaging biomarkers into routine psychiatric evaluations may help identify patients at higher risk of depression due to vascular contributions, guiding prevention and treatment strategies. These steps could provide a more comprehensive understanding of the interplay between cerebrovascular health and LLD.

Limitations

A principal limitation in our review included a language limitation, as papers not in English were not included in our search criteria. Limitations of the available research include limited shared discussion of the pathophysiology, including specific markers and proteins involved in the process of the damage and lesions that CSVD causes. Many papers offered their opinion on what can lead to LLD, but only some shared thoughts on what they believe the pathophysiology is. This is a limitation of our search results since it displays little conclusive evidence. Also, it makes it difficult to assess the validity of the papers since there is no basis or standard for what we believe may be correct.

The studies investigating the relationships between vascular disease, depression, cognition, and neuroimaging markers in aging populations have several common limitations. Many of these studies, including cross-sectional and longitudinal designs, need help establishing causality due to inherent study designs or limited follow-up durations [14,27,29]. Small sample sizes in some studies, particularly in subgroups undergoing advanced imaging, reduce generalizability and statistical power [5,9]. Additionally, some studies need more diverse populations, focusing primarily on specific age groups or clinical settings, which limits broader applicability [7,10].

Differences in methods for measuring and defining variables such as WMHs, depression severity, and cerebrovascular risk factors create challenges for comparison and reproducibility. Advanced imaging techniques, while insightful, vary in sensitivity and standardization, leading to discrepancies in measuring CBF, WMH, or arterial stiffness [5,6]. Technical differences, such as imaging techniques and analytical models, may also lead to discrepancies in reported associations [25]. Furthermore, some studies found a lack of longitudinal data to assess long-term outcomes comprehensively [4,11].

Several studies acknowledge that findings often need consistency due to potential confounding factors like vascular comorbidities, which are sometimes insufficiently controlled [12,28]. These inconsistencies emphasize the need for more rigorous study designs. Future research should employ more significant, diverse cohorts, longitudinal designs, and standardized methodologies to validate findings and clarify mechanistic pathways. Addressing these limitations would provide more definitive insights into the complex interplay between cerebrovascular health and mood disorders. Another limitation that can be highlighted in one study is the use of patient antidepressants as a confounding variable. Antidepressants alter capillary function, CBF, metabolism, and inflammation, making it difficult to determine whether observed cerebral changes are due to depression, vascular risk factors, or medication effects [11].

## Conclusions

The evidence supports an important linkage between CSVD and LLD, with ischemic changes, particularly associated with the presence of WMH, emerging as key contributors to depressive symptoms in the elderly. The reviewed studies highlighted vascular dysfunction, reduced CBF, and neuroinflammation as significant mechanisms underlying this association, supporting the VD hypothesis. This review’s findings reaffirm the importance of vascular contributions to LLD and also underscore the need for interdisciplinary approaches in geriatric mental health care. An example of a concept worth exploring is the potential relationship between HTN, resulting in small-vessel damage, and its subsequent contribution to the development of depression.

Emphasis is placed on the urgency of early detection and targeted interventions to reduce the burden of depression in aging populations. These measures may help preserve functional capacity and extend cognitive longevity in older individuals. While this study centers on the relationship between CSVD and LLD, it is important to note that ischemic events in large cerebral vessels are often more directly associated with significant depressive changes. Large-vessel ischemia, such as middle cerebral artery strokes, can cause more profound structural and functional disruptions in brain regions critical for mood regulation, including the frontal and limbic areas. This raises the possibility that large-vessel contributions to depressive symptoms may overshadow or interact with small-vessel pathology, complicating the attribution of depressive changes solely to CSVD. Avenues for future research, such as exploring novel therapeutic strategies that integrate vascular and psychiatric perspectives to optimize patient outcomes, are suggested; however, they have yet to be fully explored.
